# Noteworthy prognostic value of phospholipase C delta genes in early stage pancreatic ductal adenocarcinoma patients after pancreaticoduodenectomy and potential molecular mechanisms

**DOI:** 10.1002/cam4.2699

**Published:** 2019-12-06

**Authors:** Xin Zhou, Xiwen Liao, Xiangkun Wang, Ketuan Huang, Chengkun Yang, Tingdong Yu, Chuangye Han, Guangzhi Zhu, Hao Su, Quanfa Han, Zijun Chen, Jianlv Huang, Yizhen Gong, Guotian Ruan, Xinping Ye, Tao Peng

**Affiliations:** ^1^ Department of Hepatobiliary Surgery The First Affiliated Hospital of Guangxi Medical University Nanning Guangxi Zhuang Autonomous Region People's Republic of China; ^2^ Department of Hepatobiliary Surgery The Third Affiliated Hospital of Guangxi Medical University Nanning Guangxi Zhuang Autonomous Region People's Republic of China; ^3^ Department of Colorectal and Anal Surgery The First Affiliated Hospital of Guangxi Medical University Nanning Guangxi Zhuang Autonomous Region People's Republic of China

**Keywords:** clinical significance, early stage pancreatic ductal adenocarcinoma, molecular mechanism, pancreaticoduodenectomy, phospholipase C delta

## Abstract

The purpose of this investigation was to explore the prognostic value of phospholipase C delta (PLCD) genes in early stage pancreatic ductal adenocarcinoma (PDAC) and its potential molecular mechanisms. The prognostic value of PLCD genes in early stage PDAC was assessed using the Kaplan‐Meier method and multivariate Cox proportional hazards regression model. Genome‐wide correlation analysis was performed on PLCD3 to identify the highly correlated genes in the transcriptome. Then, PLCD3 and these correlated genes together underwent a bioinformatics analysis to elucidate the potential molecular biological functions of PLCD3 in PDAC. PLCD1 and PLCD3 are significantly overexpressed in PDAC. In PDAC patients, PLCD3 is overexpressed in certain groups of people with a history of alcoholism (*P* = .032). High expression of PLCD3 was found to be associated with lower overall survival (OS) of patients with early stage PDAC (*P* = .020; adjusted *P* = .016). A combination of PLCD3 and clinical variables was able to better predict the outcome of patients with early stage PDAC. These clinical variables are histological grade (*P* = .001; adjusted *P* = .001), targeted molecular therapy (*P* < .001; adjusted *P* < .001), radiation therapy (*P* = .002; adjusted *P* = .039), and residual resection (*P* = .001; adjusted *P* = .002). The bioinformatics analysis revealed that PLCD3 is associated with angiogenesis, intracellular signal transduction, and regulation of cell proliferation. In conclusion, PLCD3 may be a potential prognostic biomarker for early stage PDAC.

## Graphical abstract

In our investigation, PLCD1 and PLCD3 were found to be high expressed in PAAD tumor tissue compared with normal tissue. We also discovered that PDAC patients who drank alcohol had higher levels of PLCD3 than those without alcohol history. PLCD3 might be potential prognostic biomarkers for early stage PDAC according to the result of survival analysis. But in stratification analysis, PLCD3 was associated with OS only when early stage PDAC patients were in the age >60 group, female gender group, alcohol history group, low histologic grade group, with radiation therapy group, or R0 residual resection group.

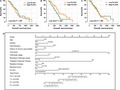

## 1 INTRODUCTION

Pancreatic ductal adenocarcinoma (PDAC) is the sixth leading cause of all cancer‐related deaths worldwide, and is predicted to become the second leading cause by 2030.^1^, ^2^ PDAC is widely known for its high malignancy and mortality, with almost equal morbidity.^3^ Although the prevalence of PDAC reported in 2018 was only 2.5% of all cancers, mortality due to PDAC was about 4.5% of all cancers.^3^ In America, PDAC‐related deaths rank fourth among all cancers in both sexes, and the incidence of PDAC has been rising steadily from 42 000 in 2009 to 57 000 in 2019.^1^, ^4^, ^5^ Since no remarkably reliable biomarkers have been identified for the detection of early stage PDAC, the low sensitivity of currently used serum tumor markers, such as carbohydrate antigen and carcinoembryonic antigen, has resulted in patients with PDAC being diagnosed predominantly at the advanced stage.^6^ Although many clinical treatment and basic research studies have been conducted during recent years, the 5‐year survival of patients with PDAC is merely 9% for all stages and 3% for patients with distant metastasis, which is the lowest survival rate among all cancers.^1^ To date, surgery is still the only method of treatment that can have a significantly positive impact on the survival of early stage PDAC patients.^6^ Therefore, exploring and identifying new therapeutic targets are vital for improving the outcome of PDAC patients.

Phospholipases, which includes phospholipase C (PLC), phospholipase D (PLD), and phospholipase A (PLA), play important roles in intracellular and intercellular signaling.^7^ Phospholipases can be stimulated by various hormones that act on receptor tyrosine kinases or G protein‐coupled receptors.^8^, ^9^, ^10^ Among the phospholipases, PLC is most widely known for its role in cell growth promotion in signaling channels.^11^ The most important and best‐known cellular signaling transduction biochemical reaction mediated by PLC is the splitting of phosphatidylinositol 4,5‐diphosphate (PIP2) into diacylglycerol (DAG) and inositol 1,4,5‐trisphosphate (IP3), which triggers the release of Ca^2+^.^12^, ^13^, ^14^, ^15^, ^16^ Both DAG and Ca^2+^ are capable of activating protein kinase C (PKC).^17^, ^18^, ^19^, ^20^, ^21^ Ca^2+^ can in turn stimulate PLC and amplify the calcium signal.^22^ In addition, IP3 is known to mediate the activation of several other protein kinases to promote/inhibit transcription in different cellular signal transduction pathways.^22^, ^23^ The lipid mediators produced by PLC regulate a variety of cellular processes that promote tumor development, including proliferation, migration, invasion, and vesicle trafficking.^11^, ^24^ PLC is the key downstream component of the EGF receptor (EGFR) that is found to be elevated in several cancers, such as breast cancer,^25^, ^26^ pancreatic cancer,^27^ and ovarian cancer.^28^, ^29^ The phospholipase C delta (PLCD) subfamily within the PLC family has three family members, *PLCD1*, *PLCD3*, and *PLCD4*.^30^, ^31^ *PLCD* genes are generally known to be tumor suppressors. *PLCD1* has been identified as a tumor‐inhibiting gene at 3p22, an area that is often mutational in esophageal cancer.^32^ In addition, decreased *PLCD1* expression is associated with a worse outcome in leukemia.^32^ It has also been reported that the proliferation, invasion, and migration of pancreatic cancer cells can be inhibited by *PLCD1*.^33^ TSPAN1 can promote PDAC cell migration and invasion by regulating the *MMP2* gene via *PLCG*.^34^ Additionally, *PLCG1* has been identified as a hub gene in PDAC through bioinformatics analysis.^35^ *PLCD3* is involved in the proliferation, migration, and invasion of nasopharyngeal carcinoma cells.^36^ Knockdown of *PLCD3* in colon cancer impairs the development of microvilli architecture, which promotes the genesis of colon cancer.^37^ However, the effect of the *PLCD* subfamily on the survival of early stage PDAC patients is still unknown. The aim of this investigation was to explore the association between *PLCD* genes and survival of PDAC patients.

## 2 MATERIALS AND METHODS

### 2.1 Data mining and processing

RNA‐Sequencing data of 182 patients with pancreatic cancer were extracted from the TCGA database (https://cancergenome.nih.gov/, accessed on 20 April 2017). In order to decrease the spread of the raw data and convert it into an expression profile, the raw data were processed using the *DESeq* package of R software (version 3.5.2; http://www-project.org). Corresponding clinical and survival data were acquired from the University of California Santa Cruz Xena Platform (UCSC Xena http://xena.ucsc.edu/, accessed at April 20, 2017). In order to better suit the purpose of the experiment and eliminate the influence of confounding factors, the patients were selected based on the following inclusion criteria: (a) histological validation; (b) early stage PDAC (pathological stage I or II, according to the 7th American Joint Committee on Cancer (AJCC)); (c) availability of complete survival data; and (d) having undergone pancreaticoduodenectomy. The inclusion criteria were applied to our previous research as well (citation). Patients with incomplete survival data, advanced‐stage PDAC, or non‐PDAC cancer patients were eliminated. The relationship between clinical variables and the outcome of the 112 early stage PDAC patients was assessed using Kaplan‐Meier analysis along with a log‐rank test.

### 2.2 Differential expression analysis of PLCD genes

Differential expression of PLCD genes between pancreatic adenocarcinoma (PAAD) tissue and normal pancreatic tissue was acquired from GEPIA (http://gepia.cancer-pku.cn/).^38^ Since PDAC accounts for 90% of PAAD, we assumed that the differentially expressed genes represent the expression state of PLCD in PDAC. In addition, we further explored the differential expression of PLCD genes in these 112 early stage PDAC patients, based on age, gender, history of alcohol consumption, tumor size, and histological classification, using the *t* test in SPSS 22.0 software. Due to the small sample size of patients at pathological stage I and patients with a history of chronic pancreatitis, the differential expression of PLCD genes in terms of pathological stage and chronic pancreatitis history was not analyzed among the 112 patients with early stage PDAC.

### 2.3 Survival analysis of the PLCD genes

Patients were divided into low expression and high expression groups, based on the median value of PLCD gene expression. Kaplan‐Meier analysis with log‐rank test was used to estimate the association between the expression levels of PLCD genes and overall survival (OS) of the patients with early stage PDAC. The OS‐associated clinical variables included histological grade, targeted molecular therapy, radiation therapy, and residual resection. A multivariate Cox proportional hazards regression model was used to adjust the survival analysis result of the PLCD genes.

A nomogram was made using the *rms* package of R software (version 3.5.2; http://www-project.org),^39^ and was used to explore the contribution of PLCD genes and several clinical variables to the OS of early stage PDAC patients. The length of the corresponding line segments represents the contribution of each variable.

After the single gene survival analysis, a combined effect survival analysis was conducted on the OS‐related gene (*PLCD3*) using OS‐related clinical variables, which included histological grade, targeted molecular therapy, radiation therapy, and residual resection. The combined effect of the OS‐related clinical variables and PLCD3 was analyzed using Kaplan‐Meier analysis with log‐rank test and the multivariate Cox proportional hazards regression model. The multivariate Cox proportional hazards regression model was used to exclude heterogeneity of the clinical variables. Details of the grouping method are shown in Table 2.

In addition, the prognostic value of *PLCD3* among patients with specific clinical characteristics was analyzed using a stratified survival analysis. We stratified patients based on their clinical characteristics and then analyzed the prognostic significance of *PLCD* isoforms in each group. Details of the stratified method are shown in Table 3.

### 2.4 Prognostic signature construction

Based on the expression of *PLCD3* and the regression coefficient derived from the result of the multivariate Cox proportional hazards regression model, a risk score was calculated for each patient, in order to construct a prognostic signature. The formula for the risk score was as follows: risk score = (expression value of gene A) × *β*_A_ + (expression value of gene B) × *β*_B_ + …(expression value of gene n) × *β*_n_; *β* meant the regression coefficient.^40^, ^41^ In addition, time‐dependent receiver operating characteristic curve (ROC) curves were generated using the *survivalROC* package of R software to test the predictive performance of the prognostic signature.

### 2.5 Genome‐wide correlation analysis and bioinformatic analysis of PLCD genes

A genome‐wide correlation analysis was performed to identify genes with a strong correlation with OS‐related (adjust *P* < .05) *PLCD3*. The first 1000 among the 31 776 genes based on correlation coefficient absolute value ranking were considered to be strongly correlated with *PLCD3*. *PLCD3* and the correlated genes were analyzed in DAVID (https://david.ncifcrf.gov/, accessed on 25 April 2019) for GO (gene ontology) terms and KEGG (Kyoto Encyclopedia of Genes and Genomes) pathway analysis.^42^ The gene‐gene relationship and protein‐protein interactions were retrieved using STRING (https://string-db.org/) 43, 44, 45 and GeneMANIA (http://genemania.org/), respectively,^46^, ^47^ while the results were visualized in Cytoscape (version 3.6.1).

### 2.6 Statistical analysis

All statistical analyses were performed using SPSS 22.0 and R 3.5.2 software. Hazard ratios (HRs) with 95% confidence intervals were used to describe the relative risk between the high and low PLCD expression groups. Kaplan‐Meier analysis with log‐rank test and the Cox proportional hazard regression model were used for the univariate and multivariable survival analysis. A *P* value of <.05 was considered to indicate statistical significance. Normalized *P* values of <.05 and a false discovery rate (FDR) of <.25 were considered to indicate statistical significance in the GSEA analysis.

## 3 RESULTS

### 3.1 Data mining and processing

The complete expression matrix output obtained using the R platform, after standardization using the DESep package, contained 31 777 gene expression values from 182 samples. Based on our objective and corresponding inclusion criteria, 70 samples without pathological confirmation of PDAC neoplasia or with incomplete survival data were removed. Data of the remaining 112 patients with pathologically confirmed PDAC and complete survival data were used for further analysis.

Among the clinical variables analyzed, targeted molecular therapy, radiation therapy, residual resection, and neoplasm histological grade were all found to be associated with OS in all 112 PDAC patients (Table S1).

### 3.2 Differential expression analysis of PLCD genes

**Figure 1:**
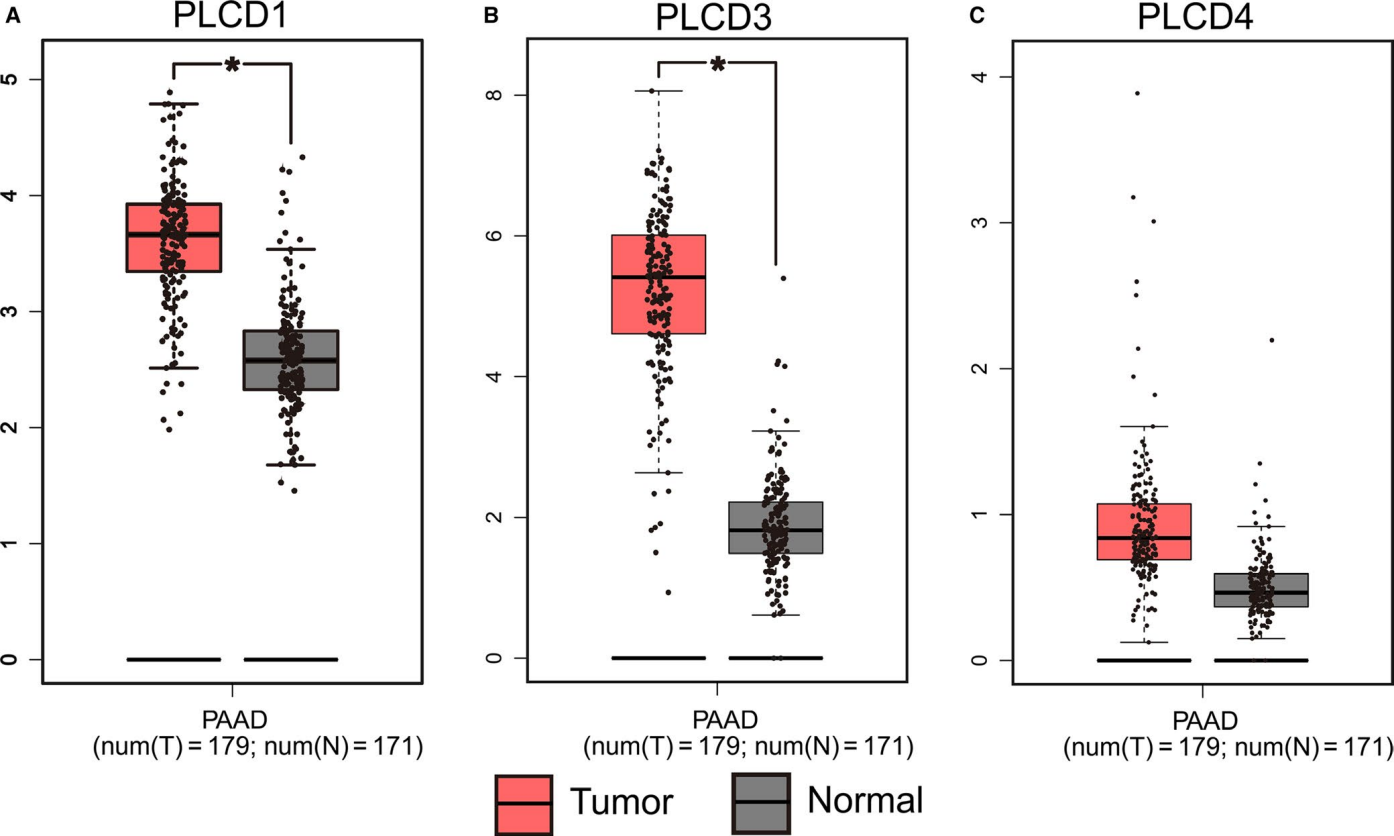
Expression of *PLCD1*, *PLCD3*, and *PLCD4* between pancreatic adenocarcinoma and normal pancreatic tissue from TCGA database. Expression of PLCD1 (A), PLCD3 (B), and PLCD4 (C)

**Figure 2:**
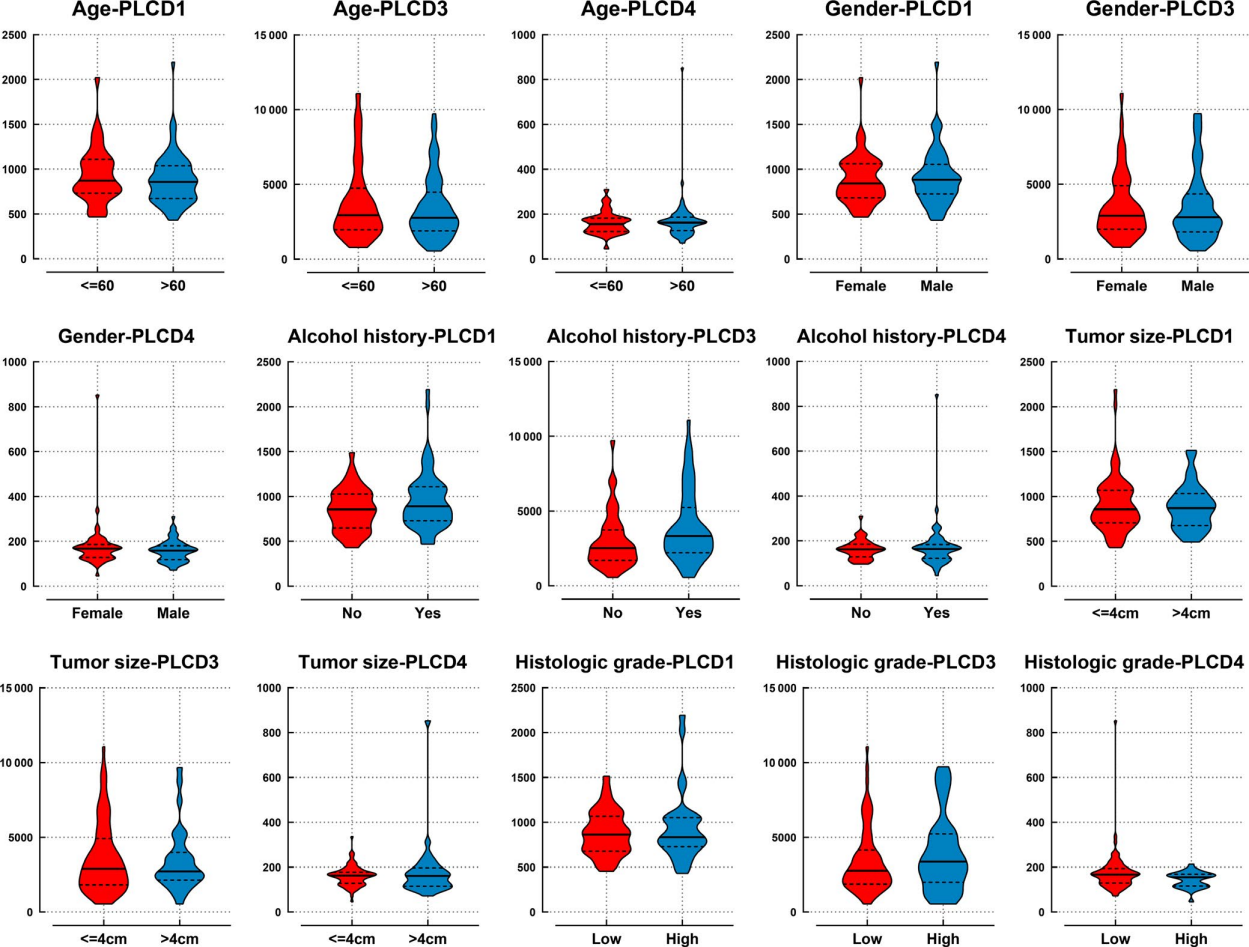
Expression of *PLCD1*, *PLCD3*, and *PLCD4* in early stage pancreatic ductal adenocarcinoma in terms of clinical variables

A boxplot retrieved from GEPIA indicated that *PLCD1* and *PLCD3* are significantly overexpressed in PAAD tissue, compared with normal tissue (Figure 1). *PLCD3* was also found to be overexpressed in patients with a history of alcoholism (*P* = .032; Figure 2H). No significant difference was found between PLCD gene expression and the other groups in terms of clinical variables.

### 3.3 Survival analysis of the PLCD genes

**Figure 3:**
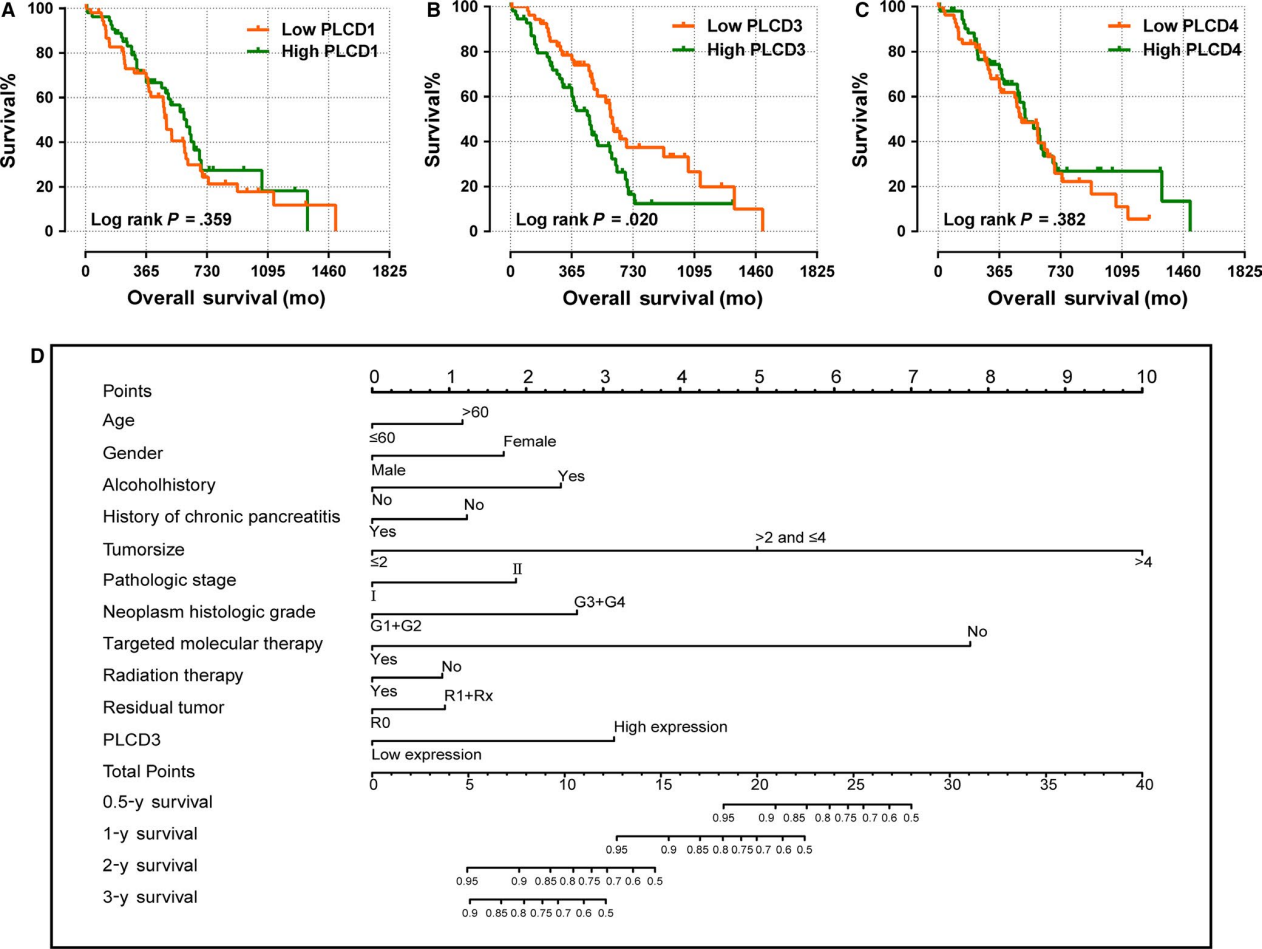
Kaplan‐Meier survival curves for PLCD genes in early stage pancreatic ductal adenocarcinoma from TCGA database (A‐C) and nomogram for predicting the 0.5‐, 1‐, 2‐, and 3‐y event (death) of patients in early stage pancreatic ductal adenocarcinoma (D)

The results of the univariate survival analysis using the Kaplan‐Meier method with the log‐rank test are shown in Table 1 and Figure 3. After adjusting for OS‐related clinical variables, which included targeted molecular therapy, radiation therapy, residual resection, and neoplasm histological grade, the results of Cox proportional hazard regression elucidated that *PLCD3* is significantly related to overall survival for the 112 PDAC patients (*P* = .020; adjusted *P* = .016) (Table 1, Figure 3B). High expression of *PLCD3* was found to be associated with shorter overall survival among the PDAC patients (adjusted HR = 1.988, 95% CI = 1.134‐3.486) (Table 1, Figure 3B).

**Table 1:** Survival analysis of PLCD genes for OS of patients in early stage PDAC

Gene expression	Patients (n = 112)	OS					
		No. of event	MST (days)	Crude HR (95% CI)	Crude Log‐rank *P*	Adjusted HR (95% CI)	Adjusted *P* a
PLCD1							
Low	56	38	485	1		1	
High	56	31	607	0.799 (0.495‐1.290)	.359	0.901 (0.527‐1.540)	.703
PLCD3							
Low	56	30	607	1		1	
High	56	39	470	1.741 (1.097‐2.935	.020	1.988 (1.134‐3.486)	.016
PLCD4							
Low	56	37	498	1		1	
High	56	32	518	0.806 (0.498‐1.306)	.382	0.745 (0.432‐1.285)	.290

Abbreviations: CI, confidence interval; HR, hazard ratio; MST, median survival time; OS, overall survival; PDAC, pancreatic ductal adenocarcinoma; PLCD, phospholipase C delta.

a Adjusted for clinical variables neoplasm histologic grade, targeted molecular therapy, radiation therapy, and residual resection.

The nomogram indicated that high *PLCD3* distinctly contributes to a worse prognosis for PDAC patients (Figure 3D).

**Figure 4:**
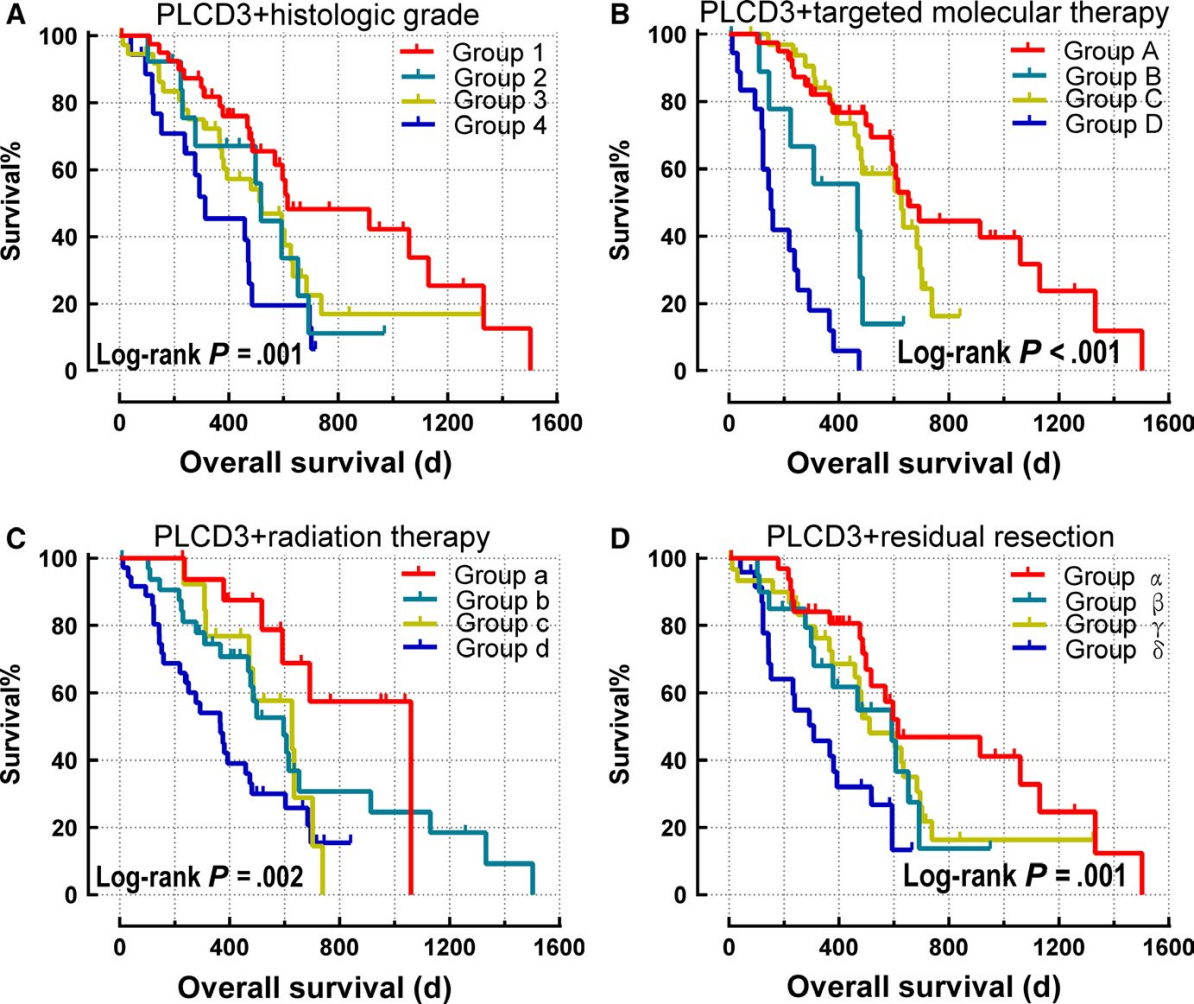
Kaplan‐Meier survival curves for joint effect of *PLCD3* and clinical variables in early stage pancreatic ductal adenocarcinoma from TCGA database. Kaplan‐Meier survival curves of *PLCD3* and histologic grade (A); Kaplan‐Meier survival curves of *PLCD*3 and targeted molecular therapy (B); Kaplan‐Meier survival curves of *PLCD3* and radiation therapy (C); Kaplan‐Meier survival curve of *PLCD3* and residual resection (D)

The combined effect survival analysis of *PLCD3* and clinical variables demonstrates that the survival time of patients with high *PLCD3* expression and a high histological grade (G3+G4) is significantly shorter than that of patients with low *PLCD3* expression and a low histological grade (G1+G2) (*P* = .001; adjusted *P* = .001; adjusted HR = 3.578, 95% CI 1.660‐7.712) (Table 2, Figure 4). The survival time of patients with high *PLCD3* expression and no targeted molecular therapy was found to be significantly shorter than patients with low PLCD3 expression and targeted molecular therapy (*P* < .001; adjusted *P* < .001; adjusted HR = 12.484, 95% CI 5.089‐30.621) (Table 2; Figure 4). Patients with high *PLCD3* expression and no radiation therapy were found to have significantly shorter survival time than patients with low *PLCD3* expression and radiation therapy, and the difference was statistically significant (*P* = .002; adjusted *P* = .039; adjusted HR = 2.778, 95% CI 1.052‐7.332) (Table 2; Figure 4), while high *PLCD3* expression and nonresidual resection resulted in shorter survival time than low *PLCD3* expression and residual resection (*P* = .001; adjusted *P* = .002; adjusted HR = 3.757; 95% CI 1.630‐8.660) (Table 2; Figure 4).

**Table 2:** Joint survival analysis of PLCD3 and clinical variables for OS of patients in early stage PDAC

Group	PLCD3	Variables	Events/total (n = 112)	MST (days)	Crude HR (95% CI)	Crude *P*‐value	Adjusted HR (95% CI)	Adjusted *P*‐value^d^
Neoplasm histologic grade								
1	Low	G1+G2	21/42	614	1		1	
2	Low	G3+G4	9/14	517	1.933 (0.866‐4.316)	.108	3.717 (1.504‐9.186)	.004
3	High	G1+G2	24/38	518	1.780 (0.966‐3.280)	.064	2.937 (1.450‐5.948)	.003
4	High	G3+G4	15/18	313	3.219 (1.607‐4.447)	.001	3.578 (1.660‐7.712)	.001
Targeted molecular therapy^a^								
A	Low	YES	22/40	652	1		1	
B	Low	NO	7/11	467	3.825 (1.549‐9.487)	.004	4.844 (1.774‐13.227)	.002
C	High	YES	19/33	627	1.528 (0.792‐2.949)	.206	1.724 (0.854‐3.480)	.129
D	High	NO	17/18	153	13.968 (6.445‐30.274)	<.001	12.484 (5.089‐30.621)	<.001
Radiation therapy^b^								
a	Low	YES	6/17	1059	1		1	
b	Low	NO	21/34	596	2.102 (0.831‐5.316)	.117	1.646 (0.597‐4.538)	.336
c	High	YES	9/13	627	2.553 (0.902‐7.228)	.078	3.135 (1.045‐9.4.4)	.041
d	High	NO	27/36	366	4.175 (1.707‐10.210)	.002	2.778 (1.052‐7.332)	.039
Residual resection^c^								
*α*	Low	R0	18/35	614	1		1	
*β*	Low	R1+RX	12/20	592	1.849 (0.858‐3.984)	.117	2.152 (0.921‐5.029)	.077
*γ*	High	R0	21/31	511	1.658 (0.855‐3.217)	.135	2.214 (1.039‐4.717)	.040
*δ*	High	R1+RX	17/24	308	3.565 (1.732‐7.338)	.001	3.757 (1.630‐8.660)	.002

Abbreviations: CI, confidence interval; HR, hazard ratio; MST, median survival time; PDAC, pancreatic ductal adenocarcinoma; PLCD, phospholipase C delta; OS, overall survival.

a Targeted molecular therapy information is unavailable in 10 patients.

b Radiation therapy is unavailable in 12 patients.

c Residual resection is unavailable in 10 patients.

d Adjusted for clinical variables neoplasm histologic grade, targeted molecular therapy, radiation therapy, and residual resection.

In the stratified survival analysis, *PLCD3* expression was found to be associated with the OS of PDAC patients who were >60 years of age, female, had a history of alcoholism, a low histological grade, had undergone radiation therapy, or R0 residual resection (Table 3).

**Table 3:** Stratification analysis of PLCD3 for OS of patients in early stage PDAC

Variables	PLCD1				PLCD3				PLCD4			
	Low	High	Adjusted HR (95% CI)	Adjusted *P*	Low	High	Adjusted HR (95% CI)	Adjusted *P*	Low	High	Adjusted HR (95% CI)	Adjusted *P*
Age (years)												
≤60	19	19	4.193 (0.939‐18.736)	.061	18	20	1.189 (0.372‐3.797)	.770	19	19	0.387 (0. 108‐1.391)	.146
>60	37	37	0.573 (0.300‐1.095)	.092	38	36	2.231 (1.169‐4.258)	.015	37	37	0.839 (0.446‐1.580)	.587
Gender												
Female	29	24	0.916 (0.418‐2.009)	.826	26	27	2.527 (1.045‐6.108)	.040	24	29	0.979 (0.440‐2.179)	.959
Male	27	32	1.248 (0.522‐2.987)	.618	30	29	1.541 (0.665‐3.571)	.313	32	27	0.677 (0.294‐1.555)	.358
Alcohol history^a^												
No	22	13	0.676 (0.271‐1.691)	.403	27	16	2.406 (0.768‐7.536)	.132	20	23	0.820 (0.312‐2.157)	.687
Yes	28	33	0.897 (0.410‐1.963)	.785	24	37	2.570 (1.057‐6.249)	.037	30	31	1.252 (0.578‐2.711)	.569
Tumor size^b^												
<=4	40	40	0.783 (0.395‐1.554)	.485	39	41	2.018 (0.94‐4.098)	.052	40	40	0.699 (0.364‐1.345)	.284
>4	15	16	0.757 (0.207‐2.768)	.674	17	14	1.466 (0.467‐4.602)	.512	16	15	1.020 (0.369‐2.822)	.970
Neoplasm histologic grade												
G1 + G2	39	41	0.633 (0.327‐1.227)	.176	42	38	2.993 (1.448‐6.188)	.003	35	45	0.988 (0.507‐1.926)	.972
G3 + G4	17	15	2.150 (0.758‐6.092)	.150	14	18	0.892 (0.275‐2.888)	.848	21	11	0.335 (0.105‐1.072)	.065
Targeted molecular therapy^c^												
No	16	13	0.847 (0.349‐2.056)	0.714	11	18	3.648 (1.164‐11.437)	.260	14	15	0.388 (0.148‐1.020)	.055
Yes	35	38	1.205 (0.598‐2.429)	0.602	40	33	1.745 (0.851‐3.574)	.128	39	34	1.087 (0.553‐2.136)	.809
Radiation therapy^d^												
No	33	37	0.834 (0.451‐1.542)	0.563	34	36	1.618 (0.842‐3.111)	.149	38	32	0.711 (0.376‐1.343)	.293
Yes	17	13	0.941 (0.269‐3.297)	0.925	17	13	4.227 (1.130‐15.814)	.032	14	16	1.108 (0.363‐3.388)	.857
Residual resection^e^												
R0	31	35	1.171 (0.587‐2.333	0.654	35	31	2.487 (1.138‐5.435)	.022	31	35	0.529 (0.245‐1.140)	.104
R1 + R2	24	20	1.197 (0.516‐2.778)	0.675	20	24	1.716 (0.701‐4.201)	.237	24	20	1.197 (0.516‐2.778)	.675

Abbreviations: CI, confidence interval; HR, hazard ratio; OS, overall survival; PDAC, pancreatic ductal adenocarcinoma; PLCD, phospholipase C delta.

a Alcohol history is unavailable in 8 patients.

b Tumor size is unavailable in 2 patients.

c Targeted molecular therapy information is unavailable in 10 patients.

d Radiation therapy is unavailable in 12 patients.

e Residual resection is unavailable in 10 patients.

### 3.4 Prognostic signature

**Figure 5:**
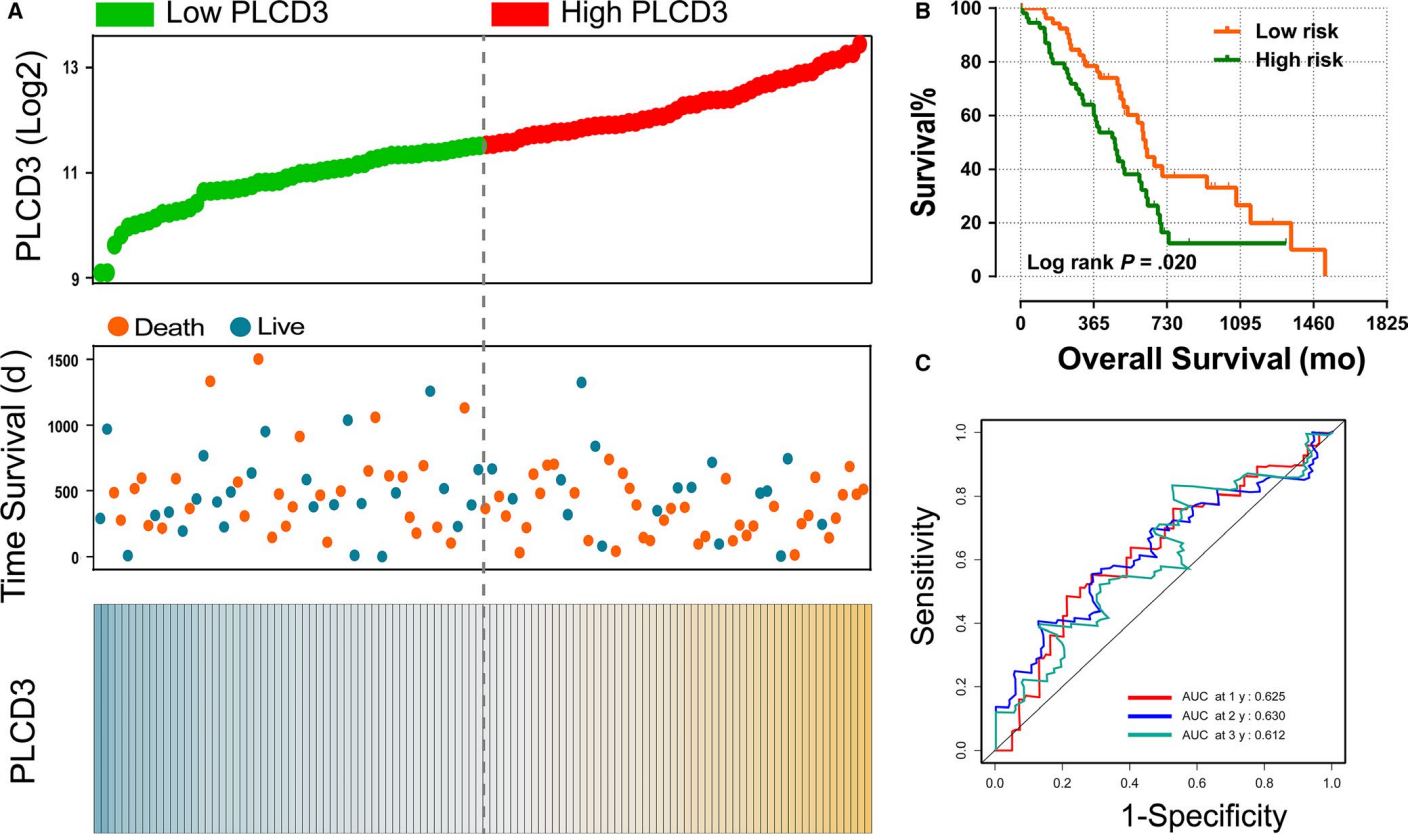
Prognostic model in early stage pancreatic ductal adenocarcinoma in terms of *PLCD3* expression. A, From up to down are risk score plot, survival status scatter plot, and heat map of the expression of *PLCD3* for low‐ and high‐risk groups. B, Kaplan‐Meier curves for low‐ and high‐risk groups. C, ROC curve for predicting 1‐, 2‐, and 3‐year survival in early stage pancreatic ductal adenocarcinoma patients by the risk score

In order to intuitively demonstrate the relationship between *PLCD3* expression and prognostic risk, and evaluate its predictive effectiveness, a prognostic signature was constructed. It included a *PLCD3* expression scatter plot, survival scatter plot, *PLCD3* expression heat map, survival curve for high‐ and low‐risk groups, and time‐dependent ROC curve (Figure 5). In this investigation, the specific formula used to calculate the risk score was as follows: risk score = (expression of MCM2) × 1.741, where 1.741 is the hazard ratio of the high *PLCD3* expression group (Table 1).

The area under the curve (AUC) of the time‐dependent ROC curve was 0.625, 0.630, and 0.612 for one‐, two‐ and three‐years survival, respectively (Figure 5C).

### 3.5 Genome‐wide correlation analysis and bioinformatics analysis of PLCD3

**Figure 6:**
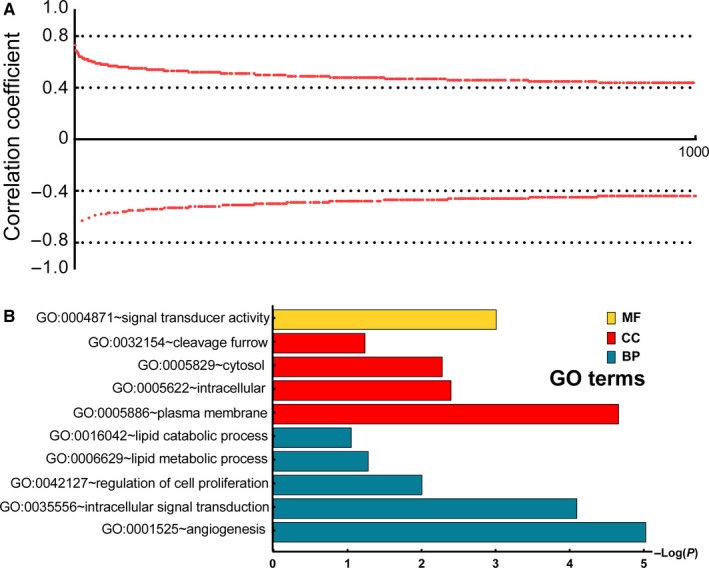
Correlation coefficient distribution of the 1000 most high correlated genes to *PLCD3* (A) and functional annotation result of *PLCD3* by KEGG pathway and GO term analysis (B)

**Figure 7:**
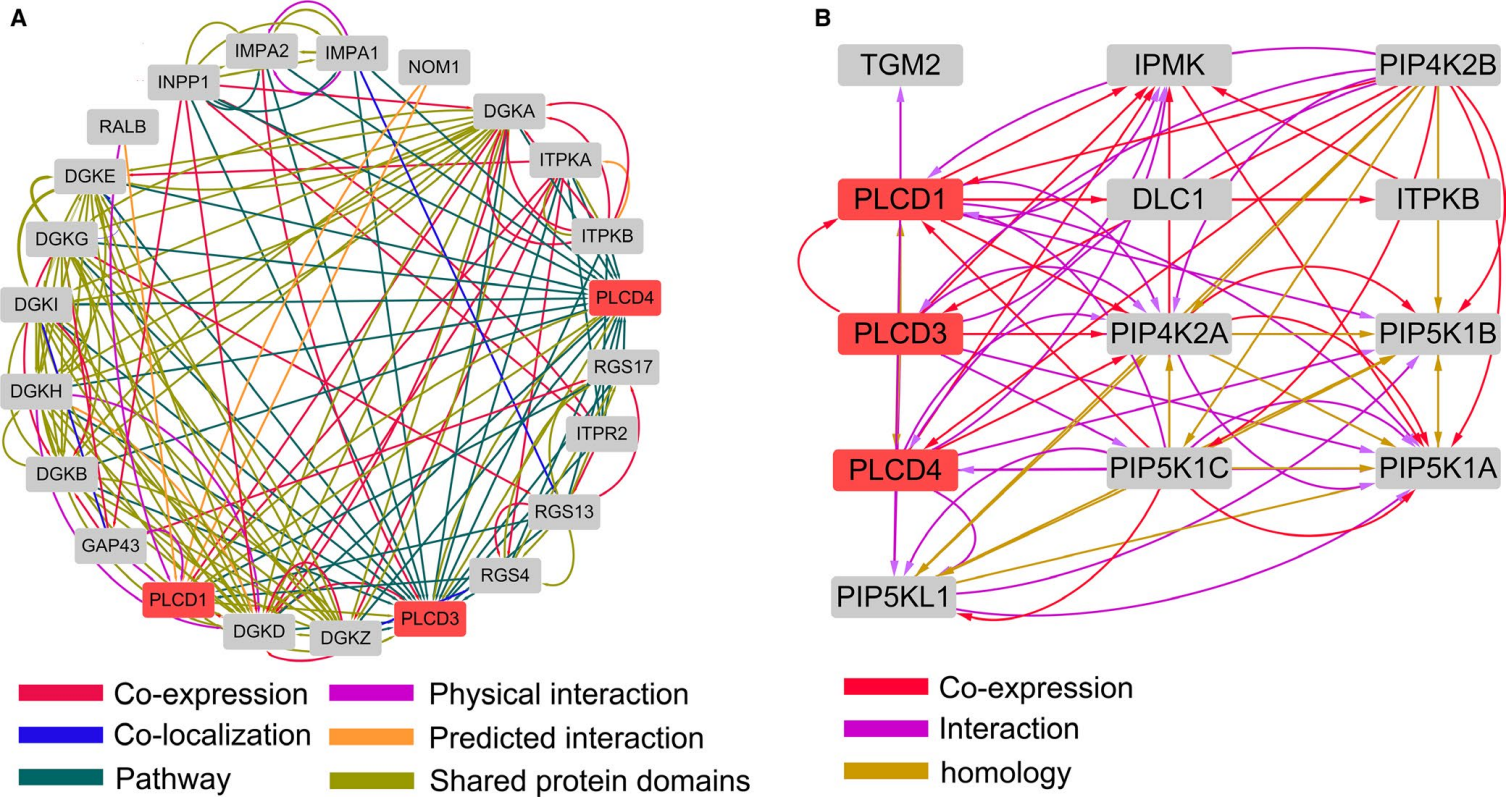
GeneMANIA gene‐gene interaction networks of the PLCD genes (A) and STRING protein‐protein association networks of the PLCD genes (B)

The correlation coefficients between the PLCD gene and strongly correlated genes (the first thousand) were all found to be greater than 0.4, with a *P* value of <.05 (Figure 6A). KEGG pathway analysis and GO annotation of these 1000 genes and *PLCD3* indicates that *PLCD3* is associated with angiogenesis, intracellular signal transduction, regulation of cell proliferation, lipid metabolic process, lipid catabolic process, plasma membrane, intracellular, cytosol, cleavage furrow, and signal transducer activity (Table S2; Figure 6B). The gene‐gene relationship network produced using GeneMANIA revealed that PLCD genes are linked to several genes of the diacylglycerol kinase (DGK) gene family, including DGKA, DGKB, DGKD, DGKE, DGKG, DGKH, DGKI, and DGKZ (Figure 7A). The protein‐protein interaction network indicates that PLCD genes are mainly co‐expressed with several phosphatidylinositol‐5‐phosphate 4‐kinase (PIP4K) subfamily and phosphatidylinositol‐4‐phosphate 5‐kinase (PIP5K) subfamily members, such as PIP4K2A, PIP4K2B, PIP5K1A, PIP5K1B, PIP5K1C, and PIP5KL1 (Figure 7B).

## 4 DISCUSSION

Although the PLCD subfamily plays an irreplaceable role in cell signal transduction, only few studies on PLCD isoforms in all types of cancer have been conducted during recent years, let alone pancreatic cancer. In this study, we found that patients with high expression of *PLCD3* have shorter overall survival and higher levels of *PLCD3* expression in tumor tissue, which indicates that *PLCD3* plays a role as an oncogene in PDAC.

It has been reported that *PLCD1* restrains the proliferation, invasion, and migration of pancreatic cancer cells CAPAN‐1 and BXPC‐3, and induces apoptosis through cell cycle inhibition at the G0/G1 phase.^48^ The prognostic significance of PLCD1 in PDAC was not revealed through this investigation, but its role as a tumor suppressor gene in breast cancer, chronic myeloid leukemia, and colorectal cancer has been widely accepted.^48^, ^49^, ^50^, ^51^, ^52^, ^53^, ^54^, ^55^, ^56^, ^57^

Although only an isoform, existing studies show that *PLCD3* functions as an oncogene. Silencing of *PLCD3* inhibits the proliferation, migration, and invasion potency of nasopharyngeal carcinoma 5‐8F cells.^36^ Similarly, *PLCD3* knockdown significantly arrests the proliferation of breast cancer mda‐mb‐231 cells.^58^ In this investigation, the high *PLCD3* expression group tended to have shorter overall survival. Taken together, it can be concluded that* PLCD3* plays a role as an oncogene in nasopharyngeal cancer, breast cancer, and pancreatic cancer. The association between *PLCD3* expression and the overall survival of early stage PDAC patients and its corresponding mechanisms observed in this study further suggest that PLCD3 may be a new target for the treatment of pancreatic cancer, and even other types of cancers, given the fact that cancers show genetic similarity during tumorigenesis.^59^

Apart from the survival analysis, we also analyzed the differential expression of PLCD. The GEPIA analysis demonstrated that *PLCD 1* and *PLCD3* are highly expressed in PAAD. Though not exactly the same, PDAC accounts for more than 90% of the histological type of PAAD.^6^ Hence, we speculated that the PAAD data could be used to analyze the expression tendency of PDAC. Moreover, we also discovered that *PLCD3* is highly expressed in PDAC patients with a history of alcoholism. This indicates that drinking alcohol may increase *PLCD3* expression levels in PDAC, which in turn increases the risk of death from PDAC. This finding corresponds with that of previous reports, which indicate that the expression of *PLCD3* increases via ethanol‐induced G‐protein activation.^60^, ^61^, ^62^

The combined effect of clinical variables and the expression of *PLCD3* indicate that patients with two risk factors have a higher hazard ratio than those with only one risk factor. In particular, patients with the combination of high PLCD expression and no targeted molecular therapy have a noteworthy high hazard ratio (HR = 12.484, 95% CI 5.089‐30.621). The stratified analysis confirmed that the prognostic value is effective in specific PDAC groups, such as those who are over 60 years old, female, have a history of alcohol use, a low histological grade, have undergone radiation therapy, or R0 residual resection.

Annotation of *PLCD3* indicates its involvement in angiogenesis, intracellular signal transduction, regulation of cell proliferation, lipid metabolism process, lipid catabolism process, plasma membrane, intracellular, cytosol, cleavage furrow, and signal transducer activity. Among these, angiogenesis, intracellular signal transduction, and regulation of cell proliferation may be the primary biological functions of *PLCD3* in PDAC, since activation of *PLCD3* can induce angiogenesis in human endothelial cells.^63^

However, our study has some shortcomings. First, the sample size used for the survival analysis was not large enough, which led to many false‐negative results regarding *PLCD1* and *PLCD4*, especially since *PLCD1* has been shown to inhibit pancreatic cancer cell growth in other studies. Meanwhile, certain clinical data and survival data values were not available, which may affect the results of the survival analysis. Second, the data used in this study were acquired from a single‐center study, and multicenter data would make the results more convincing. Third, because no transcriptome data on normal pancreatic tissues were available in the dataset acquired from TCGA, we could only use results from GEPIA to estimate the expression trend of the PLCD gene in PDAC and normal pancreatic tissues. Fourth, selection bias and recall bias may have been easily generated due to the retrospective nature of this study.

Although there are limitations in our investigation, as far as we know, this is the first study to reveal the prognostic value of *PLCD3* in PDAC. Our investigation also suggests that overexpression of *PLCD3* is associated with a history of alcoholism, which is partially explained by the role of alcohol in pancreatic cancer. The discovery of genes highly correlated with *PLCD3* opens the way forward for follow‐up research on *PLCD3* in PDAC, indicating also that the study of co‐expressed genes can aid in the discovery of the interaction between genes and explain the mechanism of PLCD in PDAC.

## 5 CONCLUSION

In our investigation, *PLCD1* and *PLCD3* were found to be highly expressed in PAAD tumor tissue, compared with normal tissue. We also discovered that PDAC patients with a history of alcoholism had higher levels of *PLCD3* than those without a history of alcoholism. Based on the results of the survival analysis, *PLCD3* may be a potential prognostic biomarker for early stage PDAC. However, the stratification analysis found that *PLCD3* is associated with OS only in early stage PDAC patients who are >60 years of age, female, have a history of alcoholism, a low histological grade, have undergone radiation therapy, or R0 residual resection.

## CONFLICT OF INTERESTS

The authors have declared that there are no competing interests.

## AUTHOR CONTRIBUTIONS

Xin Zhou and Tao Peng constructed the study design; Xiwen Liao, Xiangkun Wang, and Ketuan Huang made acquisition of data; Chengkun Yang, Tingdong Yu, Chuangye Han, Guangzhi Zhu, Hao Su, Quanfa Han, Zijun Chen, Jianlv Huang, Yizhen Gong, Guotian Ruan, and Xinping Ye made acquisition of data and made data analysis. Xin Zhou wrote the manuscript, and Tao Peng guided and supervised the manuscript. All authors read and approved the final manuscript.

## Data Availability

The datasets used and/or analyzed during the current study are available from the corresponding author on reasonable request.
