# Supplementary material for: Noteworthy prognostic value of phospholipase C delta genes in early stage pancreatic ductal adenocarcinoma patients after pancreaticoduodenectomy and potential molecular mechanisms
Source: Cancer Med. 2019 Dec 6;9(3):859–71. doi: 10.1002/cam4.2699 (PMC6997088; doi:10.1002/cam4.2699)
Supplement: Supplementary file 1 [file CAM4-9-859-s001.docx]

Table SⅠ. Basic characteristics of 112 early-stage PDAC patients^a^.

| Variables | Patients | Overall survival | | | |
| --- | --- | --- | --- | --- | --- |
|  | (n=12） | No. of events | MST (months) | HR (95% CI) | Log-rank *P* |
| Age(years) |  |  |  |  |  |
| ≤60 | 38 | 20 | 593 | 1 |  |
| >60 | 74 | 49 | 485 | 1.636(0.962-2.780) | 0.066 |
| Missing | 0 |  |  |  |  |
| Gender |  |  |  |  |  |
| Female |  | 36 | 511 | 1 |  |
| Male | 59 | 33 | 592 | 0.855(0.529-1.382) | 0.523 |
| Missing | 0 |  |  |  |  |
| Alcohol history^b^ |  |  |  |  |  |
| NO | 43 | 25 | 592 | 1 |  |
| YES | 61 | 38 | 511 | 1.276(0.765-2.128) | 0.349 |
| Missing | 8 |  |  |  |  |
| History of chronic pancreatitis^c^ |  |  |  |  |  |
| NO | 79 | 47 | 511 | 1 |  |
| YES | 9 | 7 | 607 | 1.335(0.600-2.970) | 0.478 |
| Missing | 24 |  |  |  |  |
| Tumor size^d^ |  |  |  |  |  |
| <=2 | 4 | 1 | NA | 1 |  |
| >2 and <=4 | 76 | 47 | 511 | 2.266(0.311-16.498) |  |
| >4 | 30 | 21 | 592 | 2.202(0.294-16.474) | 0.708 |
| Missing | 2 |  |  |  |  |
| Pathologic stage |  |  |  |  |  |
| Ⅰ | 8 | 4 | 236 | 1 |  |
| Ⅱ | 104 | 65 | 518 | 1.038(0.375-2.872) | 0.943 |
| Missing | 0 |  |  |  |  |
| Neoplasm histologic grade |  |  |  |  |  |
| G1+G2 | 80 | 45 | 596 | 1 |  |
| G3+G4 | 32 | 24 | 470 | 2.267(0.962-5.341) | **0.010** |
| Missing | 0 |  |  |  |  |
| Targeted molecular therapy^e^ |  |  |  |  |  |
| NO | 29 | 24 | 224 | 1 |  |
| YES | 73 | 41 | 634 | 0.168(0.095-0.296) | **<0.001** |
| Missing | 10 |  |  |  |  |
| Radiation therapy^f^ |  |  |  |  |  |
| NO | 70 | 48 | 473 | 1 |  |
| YES | 30 | 15 | 691 | 0.527(0.293-0.947) | **0.029** |
| Missing | 12 |  |  |  |  |
| Residual resection^g^ |  |  |  |  |  |
| R0 | 66 | 39 | 63 | 1 |  |
| R1+RX | 44 | 29 | 381 | 1.945(1.174-3.223) | **0.009** |
| Missing | 2 |  |  |  |  |

**Notes:** ^a^Part of data in this table also have been shown in our previous publication. ^b^Alcohol history is unavailable in 8 patients; ^c^History of chronic pancreatitis is unavailable in 24 patients. ^d^Tumor size is unavailable in 2 patients. ^e^Targeted molecular therapy information is unavailable in 10 patients, ^f^Radiation therapy is unavailable in 12 patients, ^g^Residual resection is unavailable in 10 patients.

**Abbreviations:** HR, hazard ratio; MST, median survival time; OS, overall survival; PDAC, pancreatic ductal adenocarcinoma.
