# Supplementary material for: Noteworthy prognostic value of phospholipase C delta genes in early stage pancreatic ductal adenocarcinoma patients after pancreaticoduodenectomy and potential molecular mechanisms
Source: Cancer Med. 2019 Dec 6;9(3):859–71. doi: 10.1002/cam4.2699 (PMC6997088; doi:10.1002/cam4.2699)
Supplement: Supplementary file 2 [file CAM4-9-859-s002.docx]

Table SⅡ. Bioinformatics analysis results for PLCD genes from KEGG and GO database.

| Category | Term | Count | % | PValue | -LogP | List Total | Pop Hits | Pop Total | Fold Enrichment | Bonferroni | Benjamini | FDR |
| --- | --- | --- | --- | --- | --- | --- | --- | --- | --- | --- | --- | --- |
| GOTERM_BP_DIRECT | GO:0001525~angiogenesis | 27 | 2.957284 | 9.39E-06 | 5.0274 | 760 | 223 | 16,792 | 2.675148 | 2.87E-02 | 2.87E-02 | 0.016969 |
| GOTERM_BP_DIRECT | GO:0035556~intracellular signal transduction | 37 | 4.052574 | 8.01E-05 | 4.0963 | 760 | 403 | 16,792 | 2.028549 | 0.220156 | 0.060273 | 0.144695 |
| GOTERM_BP_DIRECT | GO:0042127~regulation of cell proliferation | 17 | 1.861993 | 9.87E-03 | 2.0057 | 760 | 185 | 16,792 | 2.030327 | 1 | 0.708108 | 16.41126 |
| GOTERM_BP_DIRECT | GO:0006629~lipid metabolic process | 13 | 1.423877 | 5.27E-02 | 1.2781 | 760 | 157 | 16,792 | 1.829501 | 1 | 0.896835 | 62.42333 |
| GOTERM_BP_DIRECT | GO:0016042~lipid catabolic process | 8 | 0.876232 | 8.95E-02 | 1.0480 | 760 | 85 | 16,792 | 2.079505 | 1 | 0.94242 | 81.64938 |
| GOTERM_CC_DIRECT | GO:0005886~plasma membrane | 235 | 25.73932 | 2.19E-05 | 4.6594 | 817 | 4,121 | 18,224 | 1.271999 | 0.010828 | 0.003623 | 0.031514 |
| GOTERM_CC_DIRECT | GO:0005622~intracellular | 81 | 8.871851 | 4.00E-03 | 2.3979 | 817 | 1,332 | 18,224 | 1.356446 | 0.863581 | 0.152955 | 5.603801 |
| GOTERM_CC_DIRECT | GO:0005829~cytosol | 178 | 19.49617 | 5.26E-03 | 2.2794 | 817 | 3,315 | 18,224 | 1.197728 | 0.927099 | 0.182443 | 7.300812 |
| GOTERM_CC_DIRECT | GO:0032154~cleavage furrow | 6 | 0.657174 | 5.80E-02 | 1.2366 | 817 | 47 | 18,224 | 2.847574 | 1 | 0.532968 | 57.66749 |
| GOTERM_MF_DIRECT | GO:0004871~signal transducer activity | 21 | 2.30011 | 9.80E-04 | 3.0088 | 765 | 204 | 16,881 | 2.271569 | 0.595388 | 0.139984 | 1.52135 |

Abbreviation: PLCD, phospholipase C delta; KEGG, Kyoto Encyclopedia of Genes and Genomes; GO, Gene Ontology.
